# The Perception of Organic Food Characteristics and the Demographic and Social Profile of Consumers: A Study of the Polish Market

**DOI:** 10.3390/foods14020308

**Published:** 2025-01-17

**Authors:** Małgorzata Grzywińska-Rąpca, Mariola Grzybowska-Brzezińska, Dominika Jakubowska, Joanna Katarzyna Banach

**Affiliations:** 1 Department of Market and Consumption, Institute of Economics and Finance, Faculty of Economics, University of Warmia and Mazury in Olsztyn, 10-720 Olsztyn, Poland; malgo@uwm.edu.pl (M.G.-R.); margrzyb@uwm.edu.pl (M.G.-B.); dominika.jakubowska@uwm.edu.pl (D.J.); 2 Institute of Management and Quality, Faculty of Economics, University of Warmia and Mazury in Olsztyn, 10-719 Olsztyn, Poland

**Keywords:** organic food characteristics, plant products, animal products, demographic and social profile, relationship model

## Abstract

The aim of the research was to determine the relationship between the perception of organic food characteristics and the demographic and social profile of consumers on the Polish market. The research focused on the general characteristics and features of plant and animal products offered on the organic food market compared to conventional food. The study was conducted on a sample of 1020 respondents from different regions of Poland using structural equation modelling, which allowed for the assessment of regression and covariance relationships between variables. The models allowed an in-depth assessment of the relationships between several variables and the identification of latent factors. The results of the study showed that demographic (gender and age) and economic (income and expenditure on organic food) characteristics of Polish consumers significantly influence consumer perceptions and preferences towards organic food. Women were more likely to emphasise the importance of sensory attributes (e.g., freshness and taste), while consumers with higher incomes appreciated the organic benefits of products, especially animal products (e.g., no preservatives or hormones). Surprisingly, education was not found to be a significant differentiator in the perception of organic products. The study also provided important insights for the producers and marketers of organic food, highlighting the need to differentiate marketing strategies according to the demographic and social profile of consumers.

## 1. Introduction

International studies show that perceptions of organic food vary from country to country due to cultural and socio-economic conditions. In Germany, for example, consumers value organic certification but consider organic products to be expensive and a luxury. In Italy, on the other hand, the perception of local organic products as authentic and healthier plays a key role. In Poland, high prices and a lack of awareness limit consumer choice, although the value of organic food is increasingly recognised. Differences in the cultural and socio-economic determinants of organic food consumption indicate that perceptions of organic food are shaped by both country-specific cultural norms and socio-economic factors, such as income, education level, and availability of organic products.

Consumers perceive organic food products as healthier, more natural, and environmentally friendly, which increases their attractiveness. Consumers’ perception of organic food attributes totally depends on their demographic, social, and economic profile. These perceptions play a key role in shaping purchasing decisions and market dynamics for organic products. The importance of these perceptions is multidimensional and includes health, environmental, and economic issues, which are determined by a variety of demographic and social factors.

The awareness of organic food attributes and their perception and importance in the purchase or consumption process depend on the consumers’ age, education, income level, and lifestyle. Effective marketing and educational activities are key to building a positive perception of these products and reducing purchasing barriers such as price or availability.

The positive perception of organic food’s qualities strengthens consumer preferences and the demand for these products, which contributes to the effective action of players in the organic food market. Knowledge of the determinants of the perception of these foods, including demographic and social factors differentiating the perception of the qualities of organic food, will enable effective action to intensify a region’s sustainable development policy. This article highlights the importance of understanding the attributes of organic food products that influence consumers’ purchasing decisions. However, providing a direct assessment of the policies that drive purchase decisions is not the main focus of this study; rather, the aim is to offer an analysis of the perception of organic food attributes and the demographic, social, and economic profile of consumers. Searching for determinants of the perception of organic food attributes requires the categories of these attributes to be identified and their meaning in plant or animal products to be clarified.

Understanding the relationship between organic food perceptions and consumer profiles is crucial in order to better align marketing strategies and policies, thus supporting the development of the organic food market in Poland. The study fills a gap in the literature by providing new data on the perception of organic products in the context of the Polish market.

These conditions highlight the need for in-depth research into the perception of organic food attributes and their relationship with the demographic and social profiles of consumers in Poland. The results of research in this direction can be used by both producers and public institutions to promote sustainable consumption and adapt offers to consumer expectations.

With this in mind, a study was carried out to determine the relationship between the general characteristics and plant and animal product attributes of commercially available organic food (compared to conventional food) and the demographic and social profile of consumers in Poland. In addition, the following specific objectives were included in the study:

1. To develop a model of the relationship between the general characteristics of commercial organic foods and the demographic and socio-economic characteristics of consumers.
2. To identify regression relationships between the general characteristics of commercial organic food and the demographic and socio-economic profile of consumers.
3. To analyse the covariance between the demographic (gender and education) and socio-economic (income and food expenditure) characteristics of consumers.
4. To develop a model of the relationship between the characteristics of plant and animal products in organic food and the demographic and socio-economic characteristics of consumers.
5. To identify regression relationships between plant and animal product characteristics and the demographic and socio-economic profile of consumers.

## 2. Review of the Literature

### 2.1. Characteristics of Organic Food Products

Organic food is characterized by attributes that can be categorized into personal (private) and social (public) features, including environmental factors. Personal attributes identified by consumers of organic food include aspects such as taste, health benefits, freshness, and practical value [1,2].

An alternative perspective on the decision-making process for selecting food products by the target audience highlights the influence of product features. These attributes can be classified into three categories, sensory, instrumental, and symbolic, with each layer located either on the exterior or interior of the product. Sensory attributes such as taste, smell, colour, and appearance are related to the product’s physical qualities [3]. Instrumental or functional attributes fulfil physiological needs, including energy provision and support for cell and organ health. This category encompasses nutrient composition (e.g., carbohydrates, proteins, vitamins, etc.), ingredient quality, and the presence or absence of preservatives or additives. Other functional features such as the packaging size, ease of preparation, and storage method also fall under this group [3]. Symbolic attributes may connect consumption with status or help individuals communicate personal values, eco-friendly or ethical, to their community.

Organic food is perceived within a broader framework of environmental conservation and animal welfare, driven by a growing environmental awareness, health advocacy, and corporate social marketing efforts that enhance the image of organic products and their consumers [2]. Trust in the brand and manufacturing source plays a crucial role in purchasing decisions [3].

### 2.2. Determinants of Consumers’ Perception of Organic Food Characteristics

Consumers’ behaviour in the organic food market is the result of the attitudes, perceptions, and perceived characteristics of these foods. Consumer behaviour in the food market is undergoing dynamic changes, driven by increasing health awareness, changing social and cultural values, and technological innovations in food production and distribution. Among contemporary consumer expectations, perceptions, and preferences for food choices are complex and multifaceted, encompassing demographic/social as well as psychographic and sensory factors [4,5].

While socio-demographic factors, such as age and income, do not affect the perceptions of organic food in scheme regions, such as Greece and Sweden, they do have a greater impact in others, such as in EU markets, where geographic and cultural affiliations influence perceptions and preferences [6,7]. Generally, in the EU, the origin of organic products and awareness of certification logos are important, and different consumer groups show different perceptions of the qualities of and preferences for these foods based on these factors [7].

The perceived high cost of organic food discourages purchases despite the positive perceptions of health benefits. This is evident in the India context, where the perceived high cost of organic food results in consumers’ unwillingness to pay the higher price [8].

Social influences, including the role of social media and influencers, significantly affect consumer perceptions and purchasing behaviour, especially in such urban environments, such as Bangalore, India [8].

Distinctive attributes—sensory (e.g., taste, mouthfeel, freshness, appearance, etc.), health (e.g., the absence of chemicals, higher nutritional value, etc.) and environmental (e.g., sustainable production, animal welfare)—significantly impact organic food’s perception. Consumers often believe organic food to be healthier and more environmentally friendly, which makes these attributes key motivators for purchase; this is supported by studies from India and Europe [6,8]. In Greece and Sweden, the emphasis on health and family well-being further emphasizes the importance of personal health priorities in the purchase of organic products [6]. Positive sensory experiences enhance consumer trust and loyalty to organic food. In turn, health attributes, which are especially valued by health-conscious consumers, and environmental attributes, which also attract environmentally conscious individuals, increase the appeal of organic products. Equally important are ethical attributes (e.g., organic certification) and functional attributes (e.g., a lack of preservatives), which further enrich the perception of this food category. Social factors, such as the role of social media and influencers, also significantly shape perceptions and purchasing behaviour, especially in urban areas such as Bangalore, India [9]. As a result, marketing based on sensory, health, and organic messages, supported by effective consumer education and price alignment, can reinforce positive perceptions and increase demand for organic foods.

In recent years, the focus on the development of organic food production has been supported by some legal documents and policies from the European Commission. One of the key documents is the European Green Deal, which aims to transform the European Union’s food system into a more sustainable one, with a focus on reducing the use of chemical pesticides and fertilisers and improving animal welfare. As part of this strategy, the Farm to Fork Strategy was introduced, which aims to have 25% of the EU’s agricultural land used for organic production by 2030 [6]. Another important piece of legislation is Regulation (EU) 2018/848 of the European Parliament and of the Council on organic production and labelling, which aims to increase consumer confidence in certified organic food [10,11].

However, international studies show that consumer perceptions of these policies vary from country to country due to a combination of cultural values, regulatory environment, and consumer experience. These factors shape how individuals interpret and respond to policies and thus lead to different perceptions around the world. Countries with sectoral regulation often show lower demands for additional privacy protection; this reflects a sense of satisfaction with existing policies [12]. In the EU, different national preferences for consumer policy highlight the complexity of harmonising regulation across member states, as countries often have conflicting positions on policy effectiveness [13]. In Germany, which leads the organic market in Europe, consumers place high value on organic certification but perceive organic products as expensive and luxurious [14,15]. In Italy, on the other hand, the focus is on local organic products, which are perceived as more authentic and healthier [16]. In Sweden, ethical values and environmental awareness drive the purchase of organic food, while in Greece, health consciousness is more important [6]. Studies in Poland show that consumers increasingly perceive the value of organic food, but their choices are severely limited by high prices and a lack of awareness [17,18,19,20]. These differences suggest that perceptions of organic food are shaped by both cultural and socio-economic conditions. Cultural differences play a key role in shaping consumer perceptions. For example, countries with collectivist cultures may prioritise the well-being of the community over individual privacy concerns, while individualist cultures may emphasise individual privacy rights [12]. The literature suggests that cultural values influence not only privacy concerns, but also broader consumer behaviour, which influences how policies are received and understood [21]. Perceptions of organic food are strongly influenced by socio-economic factors, which vary across international contexts. Research suggests that while demographic factors may not be critical in some regions, socio-economic status, including education and income, shape consumer attitudes and purchasing behaviour towards organic products. Higher levels of education and income correlate with increased willingness to buy and pay for organic food, as consumers perceive greater utilitarian and hedonistic values in these products [22].

Sociodemographic factors such as age, gender, education level, and income have been identified in global studies as key to differentiating consumer attitudes towards organic food [20]. These factors, combined with beliefs, values, lifestyle, and sensory preferences, determine consumer choices [16,23,24]. In the Polish literature, the determinants of food consumers’ purchasing decisions include personal/demographic, psychosocial, socio-professional, biological, or economic conditions [24,25,26,27,28,29].

Research indicates that health, environmental concerns, product quality, and ethical considerations are key motivations for the purchase of organic food [30]. Many consumers view organic food as safer and healthier, which heavily influences their buying decisions [31]. Organic farming methods, valued for reducing pollution, conserving water, and improving soil fertility, contribute to the appeal of organic products, alongside their taste, freshness, and safety [32]. Despite their benefits, the higher prices of organic products—driven by elevated agricultural and certification costs—pose a challenge to broader market acceptance [33]. Price-sensitive consumers often weigh organic food’s value against their budgets, yet many still acknowledge its advantages [34].

Consumer behaviour surrounding organic food and knowledge-sharing on this topic are significantly shaped by social networks, peer influence, and family traditions, highlighting the role of interpersonal interactions in validating choices [35]. Perceptions of organic food as being healthier, with higher levels of vitamins, minerals, and antioxidants, stem from the avoidance of pesticides, chemical fertilizers, and GMOs during production. These perceptions strongly influence consumers, particularly those who prioritize health or manage allergies and chronic conditions such as cancer [36].

Environmental concerns also motivate consumers’ preferences for organic products. Organic farming methods are known to improve soil health, enhance biodiversity, and protect water resources. Consumers associate organic products with ethical practices such as the reduction in greenhouse gas emissions and the better treatment of animals. Organic products are also regarded as tastier, fresher, and more authentic, embodying transparency and the integrity of producers. Certification further strengthens trust and the perception of quality in organic foods [37].

Furthermore, organic food choices are linked to social values, such as supporting local farmers, gaining social prestige, and aligning with environmentally friendly trends. Selecting organic products is viewed as a form of social and ethical responsibility [38].

Cultural factors also influence purchasing behaviour. In collectivist societies such as India, consumers are more inclined to choose organic food due to social group norms and environmental consciousness. In contrast, individualistic cultures, such as in Australia, are strongly motivated by health benefits and personal gains motivators [39]. Studies have revealed that Spain, Turkey, and Colombia have differing consumer priorities; price sensitivity is a significant factor in Spain, reliable product information matters more in Colombia, and health concerns and support for local producers take precedence in Turkey [38].

Cultural perceptions of quality further impact consumer preferences. In developed regions such as western Europe and the United States, organic food is often seen as a premium product with superior taste and status. However, in developing nations such as India, concerns about health and environmental sustainability drive consumer choices [40]. Collectivist cultures place greater emphasis on social influence and group norms, while individualistic cultures prioritise personal values and needs in purchasing organic products [41].

This study fills a research gap by focusing on the Polish organic food market. This study offers a detailed analysis of the Polish organic food market, which is characterized by other challenges, such as the high price of organic products and a lack of consumer awareness. In addition, the analysis conducted on the relationship between demographic and socio-economic characteristics and attributes of organic products comprehensively links demographic (e.g., gender and age) and socio-economic (e.g., income and food expenditures) characteristics with the perception of specific characteristics of plant and animal products. Through the use of SEM, the survey identifies both overt and covert relationships between variables, allowing for a deeper understanding of the factors influencing the perception of organic food in Poland.

Contrary to previous studies, the results show that education levels do not have a significant impact on the perceptions of organic food, which suggests that access to information may play a larger role. The study shows how attributes such as freshness, the absence of pesticides (plant products), and the absence of hormones and antibiotics (animal products) affect consumer preferences, thus representing a more detailed approach than most proposed in previous studies.

Thise study thus fills a research gap, providing unique data and insights about the Polish organic food market that can be used to better understand consumer behaviour, to design marketing strategies, and to shape policies that support support the development of the organic food market.

## 3. Hypothesis Development

### 3.1. Demographic Characteristics of Respondents

The relationship between the general characteristics of organic food and the demographic characteristics of respondents, such as gender and education, has been observed by many authors. Studies suggest that specific demographic factors influence the consumer preferences and purchasing behaviour of organic food. Female consumers tend to have a more positive attitude towards organic food, as evidenced by studies showing higher purchase intentions among women [42]. In Serbia, women have been identified as the most frequent purchasers of organic products, especially fruit and vegetables [43]. Thus, the following hypothesis was made:

**H1.**  *There is a significant relationship between the general characteristics of organic food (taste, freshness, and a lack of preservatives) and the demographic characteristics of respondents, with gender being a stronger differentiating factor than education level.*

### 3.2. Economic Characteristics of Respondents

The relationship between organic food’s general characteristics and the respondents’ socio-economic characteristics, i.e., income, food expenditure, and organic food expenditure, provides insights into consumer behaviour. Higher consumer income is associated with a higher likelihood of purchasing organic food, as consumers with more disposable income can afford the higher prices often associated with these products [44,45]. Consumers who spend a higher proportion of their budget on food are more likely to purchase organic products, reflecting the prioritisation of health and sustainability in their food choices [45,46]. A significant proportion of consumers perceive organic food as expensive, which may discourage purchases, particularly among lower-income groups [44]. Based on the above, the following hypothesis was formulated:

**H2.**  *There is a significant relationship between the general characteristics of organic food (taste, freshness, and a lack of preservatives) and the economic characteristics of respondents (income, food expenditure, and organic food expenditure).*

### 3.3. Characteristics of Organic Food and Demographic/Economic Profile of Consumers

The complex relationship between the characteristics of plant-based products in organic foods and the demographic and social profile of consumers depends on factors such as gender, age, education, and income. Research shows that certain demographic groups’ different preferences and purchasing patterns for organic products can be used to form marketing strategies and product offerings. According to [46,47], women are more likely to buy organic food because of health concerns and environmental awareness. According to [48], older consumers prioritise organic sources and are more frequent purchasers, while younger consumers may be more influenced by social media marketing [48]. Increased awareness and knowledge of organic products correlates with higher education levels, which leads to a higher likelihood of purchase [49]. Consumers with higher incomes show more interest in organic certification and are more willing to pay higher prices for organic products [48,49]. Health and environmental concerns are therefore important in the context of organic food consumption. Consumers’ awareness of health and environmental concerns significantly influences their purchasing decisions [49]. Based on the above, the following hypothesis was formulated:

**H3:**  *There is a significant relationship between the characteristics of plant products in organic food and the demographic/social profile of consumers.*

**H4:**  *There is a significant relationship between the characteristics of animal products in organic food and the demographic/social profile of consumers.*

## 4. Methods

The statistical analysis of data obtained involved determining the relationship between the characteristics of plant and animal products offered for sale in organic food shops (compared to conventional food) and the demographic and social profile of consumers. This analysis was conducted on 1020 respondents living in different regions of Poland. The selection of respondents took into account demographic and socio-economic diversity, which allowed the study population to be representative. The CAWI method was chosen for gathering data because of its speed, the complete anonymity of the respondents, and the possibility of reaching a wide audience. Purposive sampling was used to identify the group of organic food consumers, and they were asked if they had consumed organic food. The statistically calculated minimum sample size was N = 265 (with a 95% confidence level and ±5% error). With a larger number of respondents (N = 1020), the standard error of the estimate decreased, which meant that the survey results were more accurate. Sampling (1020) therefore increased the likelihood of different groups having better representation (e.g., gender, education, etc.) and improved the accuracy of the results. A sample size of N = 1020 gave greater flexibility in comparing results between groups, which increased the analytical value of the survey and helped to minimise the risk of error that could arise from incomplete responses, refusals to participate, or incorrect responses.

The study took into account demographic characteristics, i.e., gender and education, while consumers’ socio-economic characteristics included average monthly net household income and average monthly expenditure on food (in general) and organic food. The general characteristics of organic food (compared to conventional food) included the following: no preservatives, little processing, clean environment production, a good and natural taste, an attractive appearance, a higher protein content, a short shelf life, and the use of a non-polluting production method. A detailed analysis of the characteristics of organic food and plant products took into account whether there was higher vitamin and mineral salt content, whether they were produced without artificial fertilisers, whether they were produced without plant protection products, and freshness characteristics (firmness, juiciness, etc.). Organic foods of animal origin were assessed for characteristics such as no added hormones and antibiotics, little fat, and short shelf life.

Structural equation modelling (SEM) allows complex relationships between multiple variables to be explored simultaneously, and it was also used to analyse the relationships between the variables adopted [50,51,52]. Constructed SEM models were developed with the data from a survey questionnaire that included questions on the consumption and perceptions of organic food. The goodness-of-fit indices of the proposed model constructs showed that the models can be useful in mapping reality and synthetically explaining relationships between variables. SEM modelling is the compilation of multiple regression and factor analysis. In addition to identifying regression relationships (in relation to observable variables), it allows us to identify latent factors and assess whether exogenous variables have been well selected for analysis. The IBM SPSS AMOS v.29 package was used.

## 5. Results

A key element of the study is the examination of the relationship between the general characteristics of organic food and its specific characteristics, both for products of plant and animal origin, in relation to the demographic and social profile of Polish consumers. Understanding these relationships allows for a better understanding of consumer preferences and choices, which play an important role in shaping the organic food market in Poland. General characteristics of organic food such as perceived quality, nutritional value, and environmental impact are often perceived by consumers as key factors in their choice of such products [53,54]. On the other hand, specific characteristics of plant products (e.g., freshness, taste, and organic certification) and animal products (e.g., farming methods and the absence of antibiotics) may influence purchasing decisions differently depending on the gender, age, education level, or economic situation of consumers.

An analysis of demographic and social profiles, taking into account factors such as income, food expenditure, and interest in organic food, makes it possible to identify the target groups most likely to be driven by health, environmental, or economic motivations in their choices. The results of this type of research are not only important for companies involved in the production and sale of organic food, but also for policy makers who develop strategies to support the development of sustainable agriculture and the popularisation of organic food in society.

In the context of dynamically changing consumer trends and increasing public environmental and health awareness, such research can also contribute to a better understanding of the challenges facing the organic food market. Verifying these relationships is therefore an important step in building a comprehensive understanding of Polish consumer behaviour [55,56,57].

Five hypotheses were formulated in the study and used to analyse the relationship between the demographic (such as gender and education) and socio-economic (income and expenditure on food, including organic food) characteristics of the respondents in detail.

The analyses are presented here to identify the main determinants of Polish consumers’ choice of organic food and to show the extent to which demographic and socio-economic characteristics influence consumer preferences for different categories of organic products. These results can serve as a basis for formulating marketing strategies and designing policies to support the development of the organic food market in Poland.

**Figure 1:**
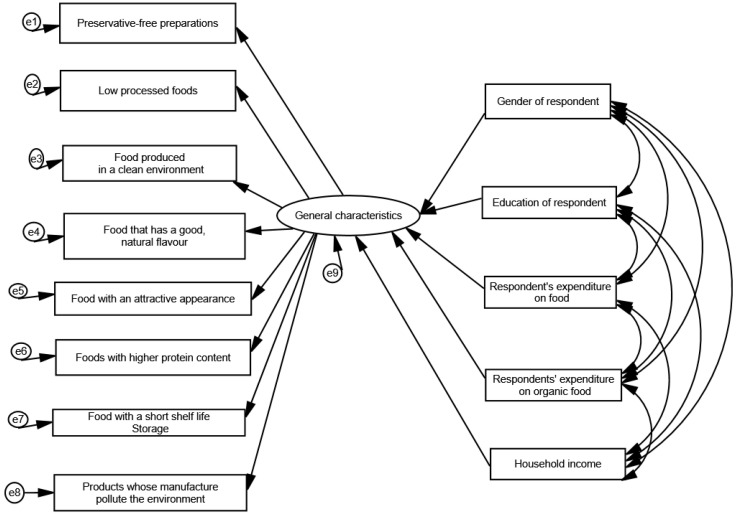
Model of the relationship between the general characteristics of commercially available organic food (compared to conventional food) and the demographic and socio-economic characteristics of the respondents. χ^2^/df = 4.716; *p*-value = 0.000; CMIN/DF = 2.716; RMSEA = 0.094; NFI = 0.878; CFI = 0.889.

The presented analysis of the survey results, with special attention paid to the verification of the model (Figure 1) and the relationship between observable and latent variables, concerns the discussion of the results and the verification of the research hypotheses. One of the aims of the survey was to determine whether the proposed model (Figure 1), assuming the existence of the constructs obtained, realistically represents the actual relationships between observable and latent variables.

The values of the indices obtained through structural modelling, which are measures of the quality of fit of the model to the data, indicate a correctly constructed dependency model. The obtained measure of the quality of fit of the model χ^2^/df is 4.716, which is lower than the upper limit for well-fitted models, is set at 5. The obtained value of χ^2^/df can therefore be considered acceptable. Among the other measures of fit, the RMSEA coefficient is highly valued by analysts; it is a measure of how poorly a model is fitted, taking into account the number of parameters that need to be estimated. Therefore, the closer its value is to 0, the better the model fit. In the case described here, the value of the RMSEA was 0.094. Another measure of the model fit is the normalised NFI fit index. This measure takes values in the range of [0, 1]. The closer its value is to 1, the better the model fit. The NFI value for the model described was 0.878, which should be considered a very satisfactory result. However, this is an index that depends on the sample size. As a result, a so-called normalised comparative fit index—CFI—was calculated. This also takes values in the interval [0, 1], and the closer it is to 1, the better the model fit.

The index was 0.889 for the model tested, which is also satisfactory. The Hoelter test was used to determine the critical value of the sample size. For the adopted significance levels of 0.05 and 0.01, the sample sizes were found to be sufficient for structural analysis. This means that, for a given significance level, the model will not be rejected if the sample size is equal to or smaller than that determined by the Hoelter test. Based on the results, it can be concluded that the sample size used to build the structural model in question is sufficient. Table 1 shows the regression coefficients and covariance values between the factors obtained by estimating the model using the maximum likelihood method.

Based on the results presented in Table 1, a statistically significant relationship was found between the general characteristics of organic food (food without preservatives; food with little processing; food with a good and natural taste; food with attractive appearance; food with attractive appearance; food with higher protein content; food with short shelf life; and products whose production pollutes the environment) and the gender of the respondent. Among the economic characteristics of the respondents, statistically significant relationships (*p* < 0.001) were observed between the general characteristics and the average monthly expenditure on organic food in the respondent’s household and the average monthly net income in the respondent’s household.

The covariate relationships in Table 2 show the relationships between the respondent’s household food expenditure and organic food expenditure, as well as between the respondent’s household average monthly net income and food expenditure (including organic food) and expenditure on food (including organic food).

**Figure 2:**
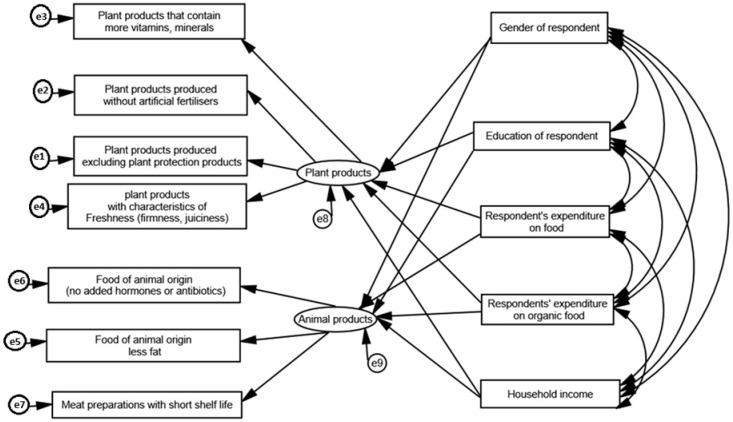
Model of the relationship between the characteristics of plant and animal products sold in organic food (compared to conventional food) and the demographic and socio-economic characteristics of the respondents. χ^2^/df = 4.259; *p*-value = 0.000; CMIN/DF = 4.259; RMSEA = 0.091; NFI = 0.843; CFI = 0.951.

In order to show the relationship between the characteristics of the respondents and those of the plant and animal products, a model was constructed taking into account these variables (Figure 2).

The values of the χ^2^ test, the RMSEA coefficient, and the NFI and CFI indices (Figure 2) testify the goodness of the model’s fit to the empirical data. The χ^2^/df measure is also a value for well-fitted models, which is favourable when considering the applicability of the model in question.

Table 3 shows the regression coefficients and covariance values between the characteristics of the plant and animal products sold in organic food (compared to conventional food) and the demographic and socio-economic characteristics of the respondents.

In the case of the model under investigation, all factors except education (relationships with plant and animal product characteristics) can be considered statistically significant and the variables next to them should remain in the model. This is shown by both the values of the critical quotients and the *p*-values obtained.

The analysis confirms the validity of the tested model, as well as the relationship between the characteristics of plant and animal products sold in organic food (compared to conventional food) and the demographic and socio-economic characteristics of the respondents. The model can be accepted on the basis of the goodness-of-fit measures presented. It can be concluded that consumers’ purchasing decisions in the organic food market are determined by gender, household net income level, and expenditure on organic food.

## 6. Discussion

The study allowed a detailed identification of the relationship between the general characteristics and plant and animal products in the organic food offer and the demographic and social profile of consumers in Poland (Table 4). The results show clear differences in consumer preferences and purchasing behaviour, which may have important implications for marketing strategies and policies supporting the development of the organic food market.

With regard to Hypotheses 1 and 2, the analysis showed that general characteristics of organic food such as taste, freshness, and a lack of preservatives were strongly related to the demographic (gender) and socio-economic (income and expenditure on organic food) characteristics of consumers. Women, in line with previous research [58], were more likely to value the sensory qualities of products due to their greater sensitivity to health and quality issues. On the other hand, men, especially in higher-income groups, paid more attention to the environmental and functional aspects of the products, in line with the findings of Bryla [19]. A surprising result is the lack of any significant effect of education on the perceptions of general characteristics, which differs from previous studies [35]. This may be due to the increasing availability of information about organic products, which reduces the importance of education as a differentiating factor in consumer preferences.

Analysis of Hypothesis 3 revealed significant covariance relationships between demographic characteristics (gender and education) and socio-economic characteristics (income and food expenditure). Women, regardless of income, more often declared choosing organic products, while men in higher-income groups mainly preferred them. In the case of plant products, consumers, especially important for women and people with higher incomes, paid special attention to their freshness, lack of pesticides, and higher nutritional value [15].

In relation to Hypothesis 4 concerning animal products, the study showed that the absence of hormones and antibiotics, as well as animal welfare, are key features for men and consumers from higher-income groups. These results are consistent with those from previous studies [59], which indicate that consumers with higher incomes are more willing to invest in premium organic products that are characterized by high quality standards (e.g., a lack of chemicals). At the same time, women, regardless of income level, indicated the importance of sensory qualities of animal products, such as taste and texture. These results are consistent with previous studies [16,60]. The developed statistical models also indicate significant relationships between demographic and socio-economic characteristics. For example, expenditure on organic food correlates with household income, and gender strongly differentiates preferences for plant and animal products. These relationships allow for practical conclusions to be drawn regarding market segmentation and directing marketing messages to target consumer groups.

✓ Messages emphasizing the sensory qualities of products (e.g., taste and freshness) should be directed mainly to women.
✓ Men and consumers with higher incomes may be interested in ecological and functional aspects, such as a lack of chemicals and animal welfare standards.

For politicians and organizations promoting organic food, the results suggest the need to increase the accessibility and education of the benefits of choosing organic products, especially in lower-income groups, which may perceive these products as being too expensive.

In addition, the results indicate that the demographic and economic profiles of consumers in Poland significantly impact the perception and choice of organic food. The findings elaborate on existing models of consumer behaviour, which suggest that gender and income level are key factors that differentiate preferences. The results confirm that gender differentiates perceptions of the sensory attributes of organic products, while higher-income consumers focus on functional and organic aspects (e.g., no preservatives and hormones). These observations are consistent with previous studies but challenge the importance of education as a significant differentiating factor. Furthermore, the results of the analyses also confirm that higher income levels correlate with a higher propensity to buy organic products. This is consistent with the theory of ’sustainable consumerism’, in which wealthier consumers see organic food as an investment in health and the environment. The study points to different factors influencing perceptions of plant (freshness and the absence of pesticides) and animal products (the absence of hormones and antibiotics), highlighting the need for further market segmentation.

In terms of practical implications, the results are significant for organic food producers, marketers, and public policy makers who aim to develop the organic food market in Poland. Producers and marketers should adapt their strategies to different consumer groups, while marketing campaigns should focus on both sensory qualities of the products, such as taste, freshness, and naturalness, and also functional and environmental aspects (e.g., the absence of hormones, positive impacts on the environment, and higher quality). The results show the need for environmental education for lower-income consumers who perceive organic food as being too expensive. Information campaigns highlight the long-term health and environmental benefits of choosing organic food. To increase market penetration, producers should aim to reduce the price of organic products or introduce support programmes for low-income consumers. Examples include loyalty schemes or government subsidies for organic products.

Taking the above into account, it should be recognized that the study provides a theoretical understanding of the dynamics of demand for organic food, taking into account local cultural and economic conditions in Poland. The study suggests that differentiation in marketing strategies and public policy, taking into account gender, income, and product specificity, can effectively reach different consumer groups and increase the awareness and availability of organic food in Poland.

For future research, the authors will continue the complex research on different regions and identified foods that are traditional, innovative, and/or introduced from abroad.

## 7. Conclusions

1. Household income and gender are key determinants of purchasing preferences among Polish consumers. Women focus on the sensory qualities of products, while men and people with higher incomes are more likely to appreciate ecological aspects.
2. Sensory attributes (taste and freshness) and ecological attributes (a lack of preservatives and animal welfare) play a key role in shaping the preferences of Polish consumers. Plant products are valued for their freshness and lack of pesticides, while animal products are valued for their lack of hormones and antibiotics.
3. Education does not significantly differentiate the perception of organic food attributes by Polish consumers, which suggests that the availability of information may be a sufficient educational factor.
4. There is a justified need to diversify the sales and promotion strategies for organic products depending on the demographic and socio-economic profile of the consumer. Women may be more susceptible to messages emphasizing the health and sensory advantages of products, while consumers with higher incomes may seek premium products with exceptional quality.
5. The accessibility of organic products for low-income Polish consumers is particularly important. Activities should include both educational campaigns and initiatives that promote the greater affordability of organic products.

## Figures and Tables

**Table 1:** Regression relationships between the general characteristics of commercially available organic food (compared to conventional food) and the demographic and socio-economic characteristics of the respondents.

Variables	Relation	Variables	Estimate	S.E.	C.R.	*p*
General characteristics	<-->	Gender of respondent	0.149	0.029	5.120	***
General characteristics	<-->	Education of respondent	0.002	0.001	1.450	0.147
General characteristics	<-->	Respondent’s expenditure on food	−0.060	0.021	−2.884	0.004
General characteristics	<-->	Respondent’s expenditure on organic food	0.000	0.000	7.463	***
General characteristics	<-->	Household income	−0.001	0.000	−10.735	***
Preservative-free preparations	<-->	General characteristics	1.000			
Hardly processed foods	<-->	General characteristics	1.053	0.049	21.633	***
Food produced in a clean environment	<-->	General characteristics	1.132	0.053	21.373	***
Food with a good natural flavour	<-->	General characteristics	1.297	0.050	25.851	***
Food with an attractive appearance	<-->	General characteristics	1.189	0.052	22.711	***
Food with higher protein content	<-->	General characteristics	1.166	0.064	18.119	***
Food with a short shelf life Storage	<-->	General characteristics	0.988	0.055	17.895	***
Products whose manufacture pollute the environment	<-->	General characteristics	1.018	0.075	13.609	***

***—*p* ≤ 0.001.

**Table 2:** Covariate relationships between demographic and socio-economic characteristics of respondents.

Variables	Relation	Variables	Estimate	S.E.	C.R.	*p*
Education of respondent	<-->	Gender of respondent	1.596	0.417	3.823	***
Education of respondent	<-->	Respondent’s expenditure on food	−4.450	0.690	−6.452	***
Respondent’s expenditure on food	<-->	Respondent’s expenditure on organic food	195.775	31.049	6.305	***
Gender of respondent	<-->	Respondent’s expenditure on food	−0.016	0.033	−0.482	0.629
Gender of respondent	<-->	Respondent’s expenditure on organic food	−2.813	18.676	−0.151	0.880
Gender of respondent	<-->	Household income	−24.249	9.405	−2.578	0.010
Education of respondent	<-->	Respondent’s expenditure on organic food	−1074.031	379.314	−2.832	0.005
Education of respondent	<-->	Household income	−38.893	189.651	-0.205	0.838
Respondent’s expenditure on food	<-->	Household income	−125.958	15.779	−7.983	***
Respondent’s expenditure on organic food	<-->	Household income	164,429.659	9978.229	16.479	***

***—*p* ≤ 0.001.

**Table 3:** Regression relationships between the characteristics of plant and animal products available for sale in organic food (compared to conventional food) and the demographic and socio-economic characteristics of respondents.

Variables	Relation	Variables	Estimate	S.E.	C.R.	*p*
Plant products	<-->	Gender of respondent	0.141	0.035	3.969	***
Animal products	<-->	Gender of respondent	0.131	0.036	3.626	***
Plant products	<-->	Education of respondent	−0.002	0.002	−1.115	0.265
Animal products	<-->	Education of respondent	0.002	0.002	1.057	0.291
Plant products	<-->	Respondent’s expenditure on food	−0.056	0.025	−2.190	0.029
Plant products	<-->	Respondent’s expenditure on organic food	0.000	0.000	7.254	***
Plant products	<-->	Household income	−0.001	0.000	−11.440	***
Animal products	<-->	Household income	−0.001	0.000	−8.642	***
Animal products	<-->	Respondent’s expenditure on organic food	0.000	0.000	8.172	***
Animal products	<-->	Respondent’s expenditure on food	−0.201	0.027	−7.340	***
Plant products produced without artificial fertilisers	<-->	Plant products	1.000			
Plant products produced (excluding plant protection products)	<-->	Plant products	1.095	0.048	22.667	***
Plant product that contain more vitamins and minerals	<-->	Plant products	1.172	0.051	22.983	***
Plant products with freshness (firmness and juiciness)	<-->	Plant products	1.155	0.048	23.967	***
Food of animal origin with less fat	<-->	Animal products	1.000			
Meat preparations with a short shelf life	<-->	Animal products	1.095	0.074	14.774	***
Food of animal origin (no added hormones and antibiotics)	<-->	Animal products	1.329	0.089	14.855	***

***—*p* ≤ 0.001.

**Table 4:** Conclusion of this study.

Hypothesis	Conclusion of This Study
H1: There is a significant relationship between the general characteristics of organic food and the demographic characteristics of the respondents (gender and education).	General characteristics of organic food, such as taste, freshness, and a lack of preservatives, were strongly associated with the gender of the consumer.
H2: There is a significant relationship between the general characteristics of organic food and the economic characteristics of the respondents (income, food expenditure, and organic food expenditure).	The general characteristics of organic food, such as taste, freshness, and a lack of preservatives, were strongly related to consumers’ economic characteristics (income and expenditure on organic food).
H3: There is a significant relationship between the characteristics of plant products in organic food and the demographic/social profile of consumers.	There were significant covariate relationships between consumers’ demographic characteristics (gender and education) and consumers’ economic characteristics (income and food expenditure).
H4: There is a significant relationship between the characteristics of animal products in organic food and the demographic/social profile of consumers.	The absence of hormones and antibiotics, as well as animal welfare, were key features for higher-income organic food consumers.

## Data Availability

The original contributions presented in this study are included in this article. Further inquiries can be directed to the corresponding author.
